# Green Synthesis of Stable Nanocomposites Containing Copper Nanoparticles Incorporated in Poly-N-vinylimidazole

**DOI:** 10.3390/polym13193212

**Published:** 2021-09-22

**Authors:** Alexander S. Pozdnyakov, Artem I. Emel’yanov, Svetlana A. Korzhova, Nadezhda P. Kuznetsova, Yuliya I. Bolgova, Olga M. Trofimova, Tatyana A. Semenova, Galina F. Prozorova

**Affiliations:** A.E. Favorsky Irkutsk Institute of Chemistry, Siberian Branch of the Russian Academy of Sciences, 1 Favorsky Str., 664033 Irkutsk, Russia; emelyanov@irioch.irk.ru (A.I.E.); korzhova@irioch.irk.ru (S.A.K.); nkuznetsova@irioch.irk.ru (N.P.K.); omtrof1@irioch.irk.ru (Y.I.B.); omtrof@irioch.irk.ru (O.M.T.); semjenova@irioch.irk.ru (T.A.S.); prozorova@irioch.irk.ru (G.F.P.)

**Keywords:** copper nanoparticles, poly-N-vinylimidazole, polymer nanocomposite, ascorbic acid

## Abstract

New stable nanocomposites with copper nanoparticles (CuNPs) in a polymer matrix have been synthesized by green chemistry. Non-toxic poly-N-vinylimidazole was used as a stabilizing polymer matrix and ascorbic acid was used as a reducing agent. The polymer CuNPs nanocomposites were characterized by Fourier transform infrared (FTIR) spectroscopy, ultraviolet–visible (UV) spectroscopy, transmission electron microscopy (TEM), scanning electron microscopy (SEM), atomic absorption spectroscopy (AAS), and thermogravimetric analysis (TGA). It was shown, using the dynamic light scattering (DLS) method, that the hydrodynamic diameters of nanocomposites depend on the CuNPs content and are in an associated state in an aqueous medium. The copper content in nanocomposites ranges from 1.8 to 12.3% wt. The obtained polymer nanocomposites consist of isolated copper nanoparticles with a diameter of 2 to 20 nm with a spherical shape.

## 1. Introduction

The specific properties of metals in an ultradispersed state open up wide opportunities for the creation of new effective catalysts, sensor systems, and drugs with high biological activity for use in medicine, ecology, and agriculture [1,2,3,4,5,6]. Metal nanoparticles are the objects of active study, due to their increased reactivity, interesting biological properties, small size, and ability to penetrate into the cells of the body [7,8,9,10,11,12]. Currently, nano-sized structures and copper nanoparticles in particular find ever increasing application in various fields. Nanomaterials including inexpensive metals attract attention as an alternative to rare and expensive noble metal catalysts. In addition, due to its high boiling point, copper can be used in chemical reactions at elevated temperatures and pressure, including reactions that can be carried out under microwave conditions [13,14]. Such unique properties of copper and its alloys contribute to the development of selective catalytic systems and are promising for application in catalysis, including electrocatalysis, photocatalysis, and gas-phase catalysis [15,16,17,18,19]. Scientific and practical interest in the study of the biological activity of copper nanoparticles is caused by the possibility of their use as regenerating and antibacterial drugs [20,21,22,23]. Copper plays an important role in the vital activity of the body. It has a catalytic effect on the processes of complete tissue regeneration [24]. Copper nanoparticles (CuNPs) have a protective effect against bacterial and fungal diseases with a reduced risk of developing resistance [25]. CuNPs can also be employed to decrease environmental pollution caused by synthetic fungicides. However, the synthesis of stable monodisperse forms of copper nanoparticles is difficult due to the tendency of copper to oxidate and aggregate.

The synthesis of stable nanoparticles of a given size that retain high chemical or biological activity for a long time is one of the important problems in polymer chemistry. Therefore, the search for ways to stabilize such particles is an urgent line of research. The incorporation of nanoparticles into polymer matrices is a common approach to address these problems. Polymers can screen the growing metal nanoparticles and inhibit their growth. Stable copper sols are formed in micellar aqueous solutions of hydrophilic polymers [26]. High molecular compounds such as chitosan, cellulose, arabinogalactan, etc. (natural compounds) [27,28], as well as poly-N-vinylpyrrolidone, polyacrylamide, poly-N-vinyl-1,2,4-triazole, etc. (synthetic compounds) are used as effective stabilizers of copper nanoparticles [29,30,31,32].

Poly-N-vinylimidazole (PVI) has a wide range of practically important properties and is widely employed in various fields [33,34]. A distinctive feature of polymers based on N-vinylimidazole (VI) is the presence of a pyridine nitrogen atom in the azole ring, which exhibits electron-donating properties. This offers wide opportunities for polymer modification. Such polymers effectively sorb metal ions to afford the coordination complexes possessing catalytic activity [35,36]. The most important feature of N-vinylimidazole polymers is solubility in water, due to which they are widely used in medicine. They have high physiological activity and are used as low molecular weight additives in medicines and as components of drug carriers [37,38].

In this work, the synthesis and characterization of water-soluble polymer nanocomposites with different CuNP contents using non-toxic poly-N-vinylimidazole as an effective stabilizer and ascorbic acid as an eco-friendly and natural reducing agent is reported. The interaction between polymeric modifiers and the resultant CuNPs was also investigated.

## 2. Materials and Methods

### 2.1. Materials

The initial N-vinylimidazole (99%), azobisisobutyronitrile (AIBN, 99%), copper acetate monohydrate (Cu(CH_3_COO)_2_∙H_2_O, 99.99%), ascorbic acid (99.99%) and deuterium oxide (D_2_O) were purchased from Sigma-Aldrich (Munich, Germany) and used as received without further purification. Ethanol (95%, OJSC “Kemerovo Pharmaceutical Factory”, Kemerovo, Russia) was distilled and purified according to the known procedures. H_2_O was used as deionized. Argon (BKGroup, Moscow, Russia) with a purity of 99.999 was used in the reaction.

### 2.2. Synthesis of Poly-N-vinylimidazole

N-Vinylimidazole (1.5 g; 16.0 mmol), AIBN (0.018; 0.1 mmol), and ethanol (1.0 g) were placed in an ampoule. The glass ampule was filled with argon and sealed. Then the mixture was stirred and kept in a thermostat at 70 °C for 30 h until the completion of polymerization. A light-yellow transparent block was formed. Then the reaction mixture PVI was purified by dialysis against water through a cellulose membrane (Cellu Sep H1, MFPI, Seguin, TX, USA) and freeze-dried to give the polymer. PVI was obtained in 96% yield as a white powder. Further, the obtained polymer was fractionated, and the fraction with M_w_ 23541 Da was used for the subsequent synthesis of the metal polymer nanocomposites.

### 2.3. Synthesis of Nanocomposites with Copper Nanoparticles

The synthesis of copper-containing nanocomposites was carried out in a water bath under reflux. PVI (5.3 mmol) and ascorbic acid (1.3–10.6 mmol) in deionized water were stirred intensively and heated to 80 °C. Argon was passed for 40 min. Then, in an argon flow, an aqueous solution of copper acetate monohydrate (0.4–3.3 mmol) was added dropwise for 3 min. The mixture was stirred intensively for another 2 h. The reaction mixture was purified by dialysis against water through a cellulose membrane and freeze-dried. Nanocomposites were obtained as a maroon powder in 83–85% yield. The copper content varied from 1.8 to 12.3 wt%.

### 2.4. Characterization

Elemental analysis was carried out on a Thermo Scientific Flash 2000 CHNS analyzer (Thermo Fisher Scientific, Cambridge, UK). FTIR spectra were recorded on a Varian 3100 FTIR spectrometer (Palo Alto, CA, USA). ^1^H and ^13^C NMR spectra were recorded on a Bruker DPX-400 spectrometer (^1^H, 400.13 MHz; ^13^C, 100.62 MHz) at room temperature. The polymer concentrations were ca. 10 wt%. Standard 5 mm glass NMR tubes were used. A Shimadzu LC-20 Prominence system (Shimadzu Corporation, Kyoto, Japan), equipped with a differential refractive index detector Shimadzu RID-20A, was used to determine the molecular weight of the polymer by gel permeation chromatography. An Agilent PolyPore 7.5 × 300 mm (PL1113-6500) column was used and chromatographic analysis was performed at 50 °C, with used *N*,*N*-dimethylformamide (DMF) as the eluent at a flow rate of 1 mL/min. The samples were dissolved for 24 h with stirring at 50 °C. Calibration was performed using a set of polystyrene standards, consisting of 12 samples with molecular weights ranging from 162 to 6,570,000 g/mol (Polystyrene High EasiVials PL2010-0201).

The copper content was estimated by atomic absorption analysis using a Shimadzu AA-6200. Microwave digestions were performed in a closed microwave oven system (CEM Corporation Mars 5, Matthews, NC, USA). The optical spectra of the nanocomposites were studied on a Shimadzu UV-2450 spectrophotometer (Shimadzu Corporation, Kyoto, Japan). Microphotographs were obtained using a transmission electron microscope (Leo 906E, Zeiss, Oberkochen, Germany).

Thermogravimetric analysis and differential scanning calorimetry were performed on an STA 449 Jupiter (Netzsch, Germany) at a heating rate of 5 °C per min from 20 to 1150 °C in an air atmosphere. The sample weight was 7 mg. Analysis of the qualitative and quantitative composition of the evolved gaseous thermolysis products was performed using a QMS 403 C Aeolos quadrupole mass spectrometer (Netzsch, Selb, Germany) coupled with the thermal analyzer.

The prefiltered deionized water or water–salt NaNO_3_ (0.01 mol/L) solution with 0.1 mg/mL PVI and nanocomposites concentration was applied to determine the hydrodynamic particle diameter of the studied samples by means of the dynamic light scattering (DLS) method using a ZetaPALS Zeta Potential Analyzer with a BI-MAS module (Brookhaven Instruments Corporation, Holtsville, NY, USA). The measurements were carried out in thermostated cuvettes with an operating temperature of 25 °C and an angle of detection of scattered light equal to 90 °C. The surface structure and EDX were studied by a FEI Company Quanta 200 (Hillsboro, OR, USA) scanning electron microscope with an EDAX X-ray microanalysis attachment with a nitrogen-free cooling GENESIS XM 2 60-Imaging SEM with APOLLO 10. The sample was fixed on a substrate with double-sided scotch tape and coated with gold in a SDC 004 vacuum unit (OERLIKON BALZERS, Balzers, Liechtenstein).

The electrical conductivity of the synthesized polymers was measured by impedance spectroscopy at 25 °C and a relative humidity of 40% on a PARSTAT 2273 electrochemical workstation (Princeton Applied Research, Oak Ridge, TN, USA).

## 3. Results and Discussion

### 3.1. Polymer of N-vinylimidazole

**Scheme 1:**
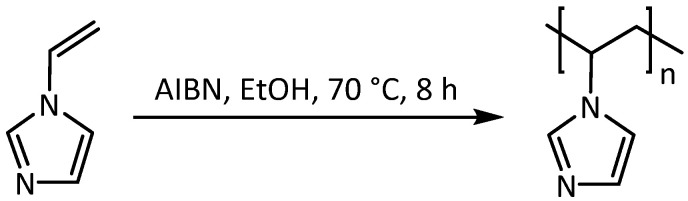
Synthesis of poly-N-vinylimidazole.

Radical polymerization of N-vinylimidazole was carried out in ethanol in the presence of an initiator (AIBN) at 70 °C in an argon atmosphere. The polymerization proceeds as shown in Scheme 1.

**Figure 1:**
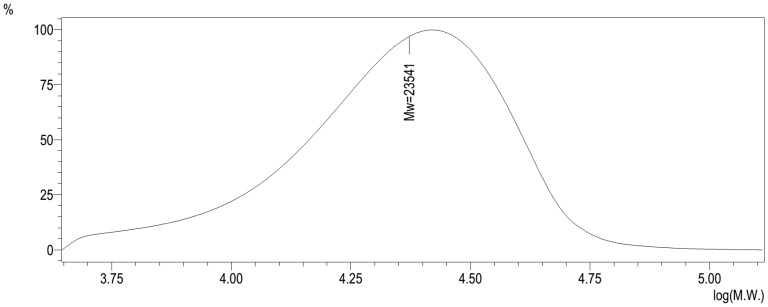
GPC traces of PVI were used to obtain nanocomposites.

The obtained poly-N-vinylimidazole was fractionated from ethanol solution by fractional precipitation, using acetone and hexane as precipitants. Seven fractions with different molecular weights were isolated, containing from 8 to 57% of the initial polymer weight. The molecular weight characteristics of the obtained fractions were determined using GPC. The fraction with the maximum yield was used as a stabilizing polymer matrix to obtain copper-containing nanocomposites. The measured M_n_ and M_w_ values of the PVI fraction used were 18,325 and 23,541 Da, respectively. The polymer showed a unimodal molecular weight distribution (Figure 1). The polydispersity index (M_w_/M_n_) of the polymer was 1.28. The synthesized PVI is soluble in water and bipolar organic solvents (DMF and DMSO).

**Figure 2:**
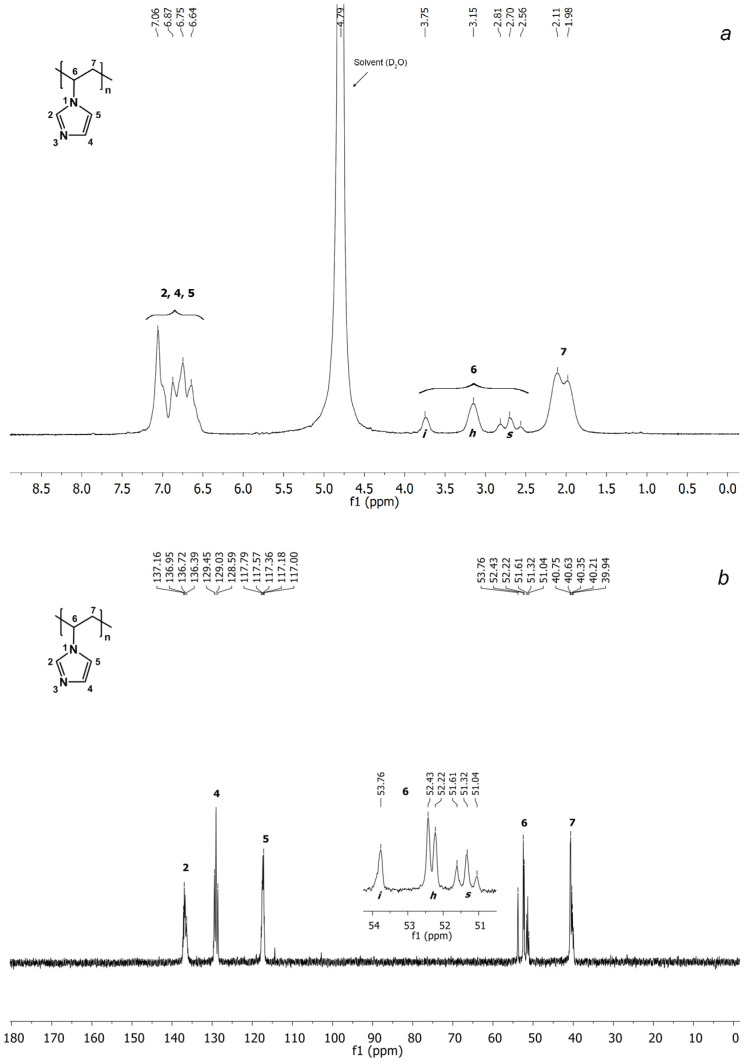
^1^H (**a**) and ^13^C (**b**) NMR spectra of PVI.

The synthesized PVI was characterized by ^1^H and ^13^C NMR analysis (Figure 2). The ^1^H spectrum of PVI contains the characteristic proton signals from the imidazole ring at 6.64–7.06 ppm (2, 4, 5). The broadened signals at 1.98–2.11 ppm (7) belong to protons of the -CH_2_- backbone groups. Previously, it was shown that the methine signal of the main polymer chain is sensitive to macromolecular chain configuration and allows the determination of polymer tacticity and ratios of different triads [39,40,41]. According to this, the methine proton signals of our sample are split into three main groupings at 2.56–2.81 ppm (triplet from the CH backbone for the syndiotactic (s) triads), at 3.15 ppm (singlet from the CH backbone for the heterotactic (h) triads), and at 3.75 ppm (singlet from the CH backbone for the isotactic (i) triads) (Figure 2). As evidenced from the character and position of these chemical shifts, PVI shows a predominantly atactic configuration (h > s > i), as did PVI synthesized by radical polymerization of VI with AIBN in methanol at 50 °C by Barboiu et al. [41]. Isotactic, heterotactic, and syndiotactic triads are in the proportions 1:5:1.5.

In the ^13^C NMR spectrum of PVI, the signals of the imidazole ring carbons are detected at 136.39–137.16 ppm (C2), 128.59–129.45 ppm (C4), and 117.00–117.79 ppm (C5) (Figure 2). The signals at δ 39.94–40.75 ppm (C7) are assigned to the methylene groups carbons of the main polymer chain. Tacticity effects also account for the appearance of the three groups of methine signals at 51.04–51.61 ppm (triplet from the CH backbone for the syndiotactic (s) triads), at 52.22–52.43 ppm (doublet from CH backbone for the heterotactic (h) triads), and at 53.76 ppm (singlet from the CH backbone for the isotactic (i) triads). 

### 3.2. Synthesis and Characterization of Polymeric CuNPs Nanocomposites

The synthesis of nanocomposites with copper nanoparticles (CuNPs) was performed by an eco-friendly, simple, and reproducible method, by the chemical reduction of copper(II) ions in the presence of PVI as a particle stabilizer. The reaction was carried out at the molar ratio of PVI:Cu(II) varied from 40:1 to 5:1 (Table 1).

Ascorbic acid, which ensures the compliance of synthetic methods with the principles of “green chemistry” and the safety of the target product, was employed as a reducing agent used [42]. The reduction of Cu^2+^ to CuNPs occurred via the transition of ascorbic acid to dehydroascorbic acid through the intermediate radical ion semidehydroascorbic acid [43]. Oxidation of ascorbic acid was catalyzed by copper ions [44]. In addition, ascorbic acid has antioxidant properties due to its ability to trap free radicals and reactive oxygen molecules [45]. Thus, ascorbic acid plays a dual role as a reducing agent for the synthesis of CuNPs and as an antioxidant for their protection.

**Scheme 2:**
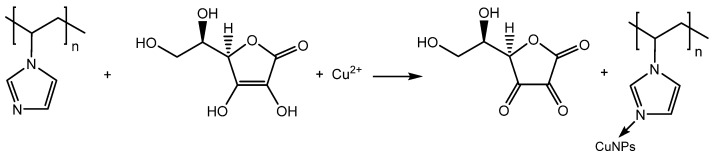
Formation and stabilization of copper nanoparticles.

The formation of copper nanoparticles was accompanied by their donor–acceptor interaction involving the imidazole ring, which acted as a ligand (Scheme 2). Coordination was provided by the electron–donor nitrogen in position three of the imidazole ring, as in the case of binding with Cu^2+^ [46].

By mixing aqueous solutions of polymer and ascorbic acid, a clear solution was obtained. After adding blue copper acetate solution to it, the color of the solution gradually changes from yellow to burgundy and finally to maroon. The appearance of a yellow color indicated the initiation of the reduction reaction. Ascorbic acid reduces the ionic form of the metal to the metallic state. A color change to maroon indicates the end of the formation of copper nanoparticles. It should be noted that the coordination interaction of copper ions with functional imidazole groups of the polymer macromolecules creates a favorable microenvironment that contributes to the effective stabilization of nanoparticles in the early stages of their formation. During the synthesis, excess ascorbic acid was required to complete reduction and avoid the oxidation of CuNPs. Additionally, in order to prevent oxidation of copper nanoparticles during synthesis, an oxygen-free environment was created.

It was found that the copper content in nanocomposites varies from 1.8% to 12.3% by elemental analysis and atomic absorption spectroscopy (Table 1). The copper content depends on the initial molar ratio of the stabilizing polymer and Cu(II). The stabilizing ability of the polymer matrix relative to a large number of formed nanoparticles decreases with an increase in the copper content relative to the polymer. This inevitably leads to partial coagulation and the formation of larger nanoparticles. An increase in the copper content above 6.7 wt% led to a partial loss of the solubility of nanocomposites **3** and **4** in water and dipolar organic solvents.

**Figure 3:**
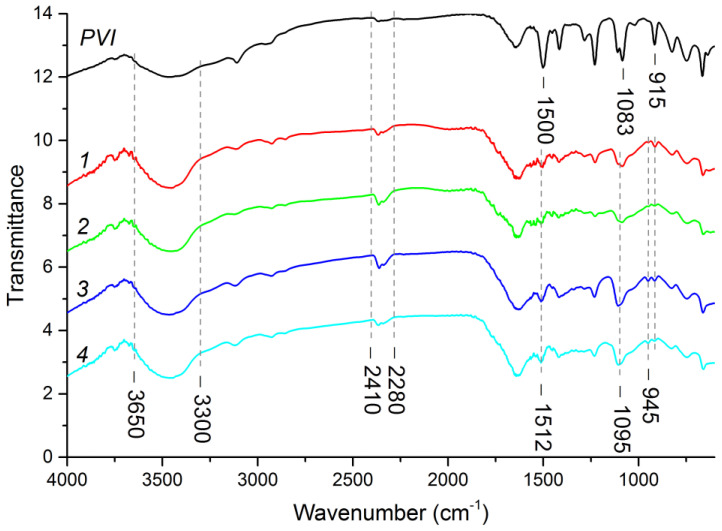
FTIR spectra of PVI and polymer nanocomposites with CuNPs **1**–**4**.

The IR spectrum of the PVI polymer contains characteristic bands of the stretching and bending vibrations of the imidazole ring at 3109 (C–H), 1500 (C–C and C=N), 2280–2410 (NH, protonated ring), between 1083 and 1286 (C–H and C–N), 915 (ring), between 745 and 826 (C–H), and 665 cm^−1^ (N–C) (Figure 3). Band vibrations at 2946 (C–H and CH_2_), 1416 (C–H or ring), and 1018 cm^−1^ (C–H and C–C) correspond to the vibrations of the main chain. The FTIR spectrum of the synthesized PVI is in good agreement with the data in the literature [47,48].

Analysis of the IR spectra shows that the obtained nanocomposites do not cause significant changes in the polymer matrix. However, the ring vibrations of imidazole at 1500, 1083 and 915 cm^−1^ are shifted to 1512, 1095, and 945 cm^−1^, respectively, upon metal nanoparticles incorporation. This indicates the coordination interaction between the copper and nitrogen atoms at position three of the imidazole ring in nanocomposites **1**–**4**. The intensity of the band at 915 cm^−1^ rises with an increase in the copper content in the nanocomposites and is clearly visible in **3** and **4**. Similar band shifts are characteristic of PVI upon complexation with metal ions [49,50]. In addition, the presence of a band at 915 cm^−1^ in all nanocomposites shows that the free imidazole groups are not involved in complexation with Cu^2+^ ions. The spectra of nanocomposites **1**–**4** contain the wide band of the protonated imidazole ring in the region of 2280–2410 cm^−1^. The broad band between 3650 and 3300 cm^−1^ is assigned to the stretching vibration of physically bound water, which indicates polymer association through intermolecular hydrogen bonds.

**Figure 4:**
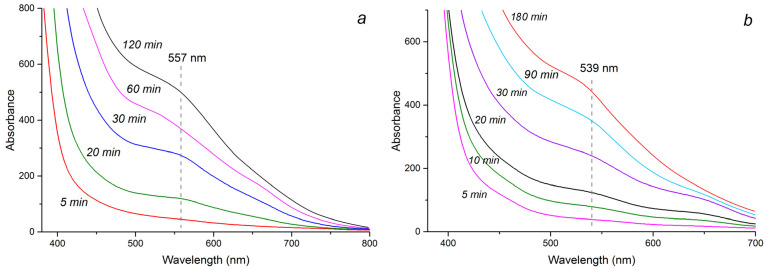
UV spectra of aqueous solutions of polymer nanocomposites **2** (**a**) and **4** (**b**).

The optical absorption spectra of the reaction solutions in an aqueous medium confirm the formation of nanosized copper particles (Figure 4).

Electronic absorption spectra of nanocomposites **1**–**4** were recorded after adding copper acetate monohydrate to a mixture of polymer and ascorbic acid at different times. The surface plasmon band with an absorption maximum in the range of 535–557 nm, caused by the collective oscillation of conduction electrons on the surface, confirms the formation of CuNPs. The formation of CuNPs in solution was observed after 20 min. The copper reduction reaction was completed after 120 min for nanocomposites **1** and **2** (Figure 4a) and after 180 min for nanocomposites **3** and **4** (Figure 4b).

The formation of Cu_2_O with plasmon absorption at 480–485 nm was not detected in the synthesized nanocomposites [51,52].

The high stabilizing ability of PVI is evidenced by the identity of the plasmon absorption band of copper nanoparticles before and after centrifugation (10,000 rpm, 15 min). The presence of a free electron pair at the N atom of the imidazole ring leads to the formation of coordination bonds between CuNPs and the corresponding interaction centers. Such an interaction provides effective stabilization of copper nanoparticles, which prevents their aggregation for a long time.

**Figure 5:**
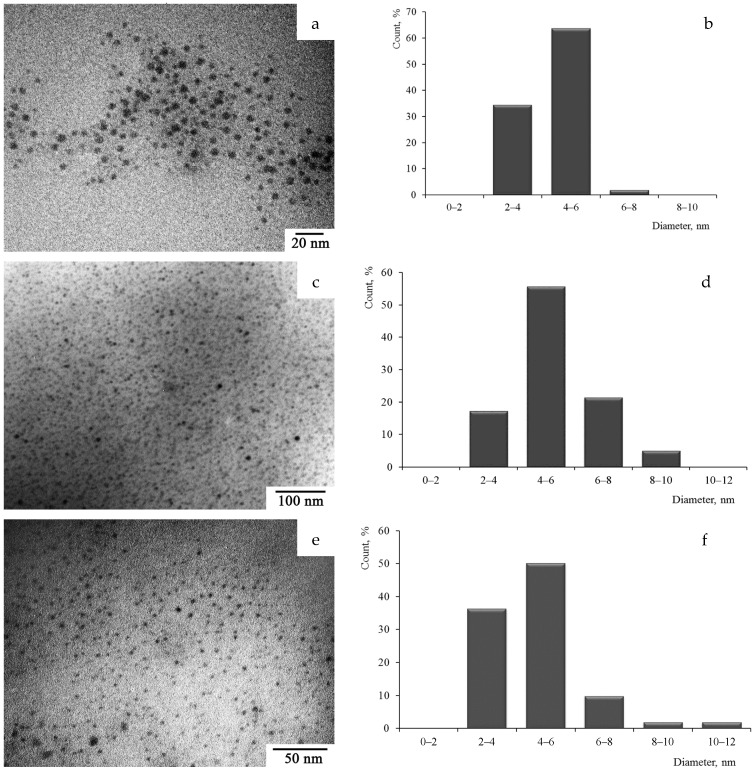

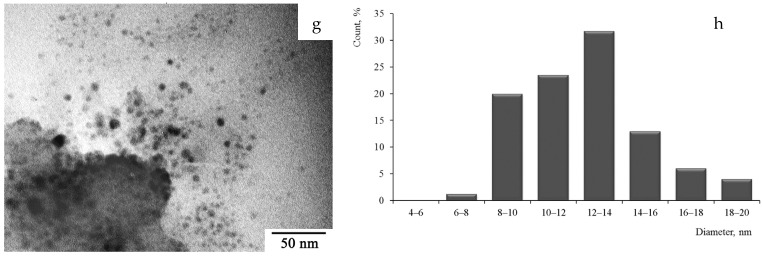
Electron microphotographs (**a**,**c**,**e**,**g**) and diagrams of CuNPs size distribution (**b**,**d**,**f**,**h**) of polymer nanocomposites: **1** (**a**,**b**), **2** (**c**,**d**), **3** (**e**,**f**), and **4** (**g**,**h**).

The shape and size of nanoparticles in nanocomposites **1**–**4**, as well as their distribution in the polymer matrix, were studied using TEM. Isolated electron contrast copper nanoparticles in nanocomposites **1**–**4** are uniformly distributed in a polymer matrix and have a predominantly spherical shape with dimensions of 2–20 nm. The copper content in the nanocomposites **1**–**4** influences the size dispersion of copper nanoparticles. The smallest size distribution of nanoparticles is observed in nanocomposite **1**, in which the poorest copper content is shown (Figure 5).

The PVI matrix loses its ability to stabilize large amounts of nanoparticles (или CuNPs) at a high copper content (nanocomposite **4**), which leads to coagulation with the formation of larger nanoparticles (Figure 5).

Number averages (*D_n_*) and weight averages (*D_w_*) diameter of nanoparticles, and polydispersity indices (*PDI*) (Table 2) were calculated based on the nanoparticle size data using the following three equations [53]:

$$\begin{array}{l} D_{n}=\frac{\sum_{i}n_{i}D_{i}}{\sum_{i}n_{i}}\\ D_{w}=\frac{\sum_{i}n_{i}D_{i}^{4}}{\sum_{i}n_{i}D_{i}^{3}}\\ PDI=D_{w}/D_{n} \end{array}$$

where *n_i_* is the number of particles of size *D_i_*.

The data in Table 2 indicate that copper nanoparticles in nanocomposites **1**–**4** have a narrow size dispersion. With an increase in the copper content in the stabilizing matrix from 1.8 to 12.3%, the sizes of nanoparticles increase by 2.9 (*D_n_*) and 3.7 (*D_w_*) times. The *PDI* of nanoparticles in synthesized nanocomposites **1**–**4** varies from 1.11 to 1.48. The maximum *PDI* is achieved for nanocomposite **3**.

The effective hydrodynamic diameters of the initial PVI and synthesized nanocomposites **1**–**4** were measured by dynamic light scattering. The histograms show that the dependence of signal intensity on hydrodynamic diameter for PVI in an aqueous medium is characterized by a monomodal distribution with a maximum at 264 nm. The scattering particle diameter is up to 10 nm, which corresponds to the M_w_ of the synthesized PVI. It can be assumed that PVI macromolecules are associated in an aqueous solution. It is found that in an aqueous–salt medium, the macromolecular associates decompose into individual polymer chains with an effective hydrodynamic diameter of 5 nm. Therefore, PVI in water forms large supramolecular structures, which are formed due to the intermolecular interaction of individual macromolecules. The formation of such associates occurs via hydrogen bonds involving the imidazole groups, which belong to different molecular chains of the polymer [54]. Since PVI in a neutral medium is in a partially protonated state (the degree of ionization is about 10% at pH 6), one can expect intermolecular interaction with the participation of protonated and non-protonated imidazole rings [54,55].

**Figure 6:**
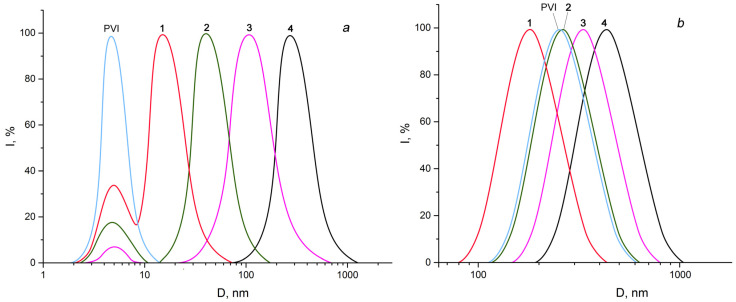
Histogram of the distribution of scattering particles over hydrodynamic diameters for PVI and nanocomposites 1–4 in an aqueous-salt solution (**a**) and in water (**b**).

It is established that the formation of copper nanoparticles in the presence of PVI leads to the formation of a dispersed phase of nanocomposites, the hydrodynamic dimensions of which are determined by the copper content (Figure 6). The histograms of nanocomposites in an aqueous–salt solution are characterized by a bimodal distribution (Figure 6a). An increase in the copper content in nanocomposites **1**–**4** (Table 1) is accompanied by a growth of the average hydrodynamic diameters of macromolecular coils from 17 to 290 nm. Macromolecular coils of the initial PVI are observed only at a large excess of polymer (nanocomposites **1**–**3**). Their intensity decreases with increasing copper content. This indicates the presence of PVI, which is not involved in the stabilization of copper nanoparticles.

Aqueous solutions of nanocomposites are characterized by a monomodal distribution of scattering particles (Figure 6b). The average hydrodynamic diameter of macromolecular coils increases from 193 to 445 nm with an increase in the metal content in nanocomposites.

In nanocomposites **1**–**3** scattering PVI particles not involved in stabilization of CuNPs are not observed. This indicates that they are in an intermolecular association with macromolecular coils of nanocomposites. Association suppression in an aqueous salt solution leads to good separation of the mixture of individual macromolecular coils of nanocomposites and free PVI. This allows us to determine the true size of the macromolecular coils of nanocomposites.

**Figure 7:**
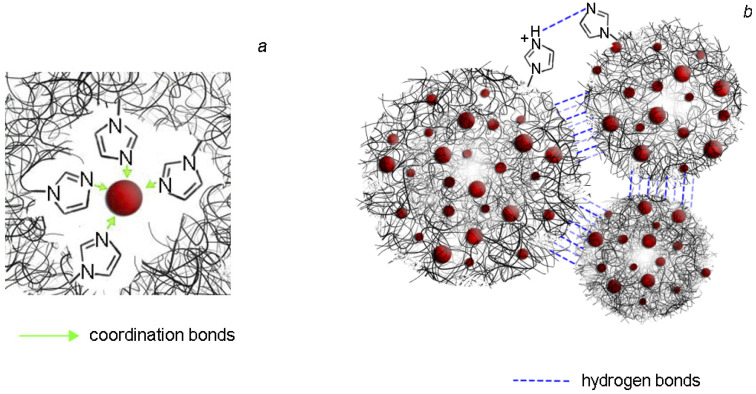
Stabilization of CuNPs by PVI (**a**) and association of nanocomposites by hydrogen bonds (**b**).

Thus, nanocomposites are macromolecular coils consisting of CuNPs in the PVI stabilizing matrix. The interaction between the components is provided by the coordination bonds of imidazole rings with copper atoms on the surface of nanoparticles (Figure 7a). In this case, the resulting bond of nanoparticles with PVI will be significantly enhanced by cooperative multipoint coordination bonding simultaneously with many surface atoms. An increase in the content of CuNPs in nanocomposites leads to an increase in the diameter of macromolecular coils. This indicates the intermolecular crosslinking of individual PVI macromolecules by nanoparticles, which act as the coordination crosslinking agent. In an aqueous solution, nanocomposites **1**–**4** are supramolecular structures consisting of individual macromolecular coils of nanocomposites saturated with CuNPs, which are associated with each other due to hydrogen bonds between imidazole groups (Figure 7b).

**Figure 8:**
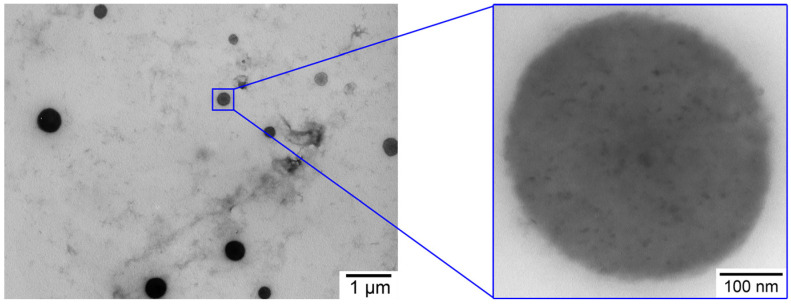
Electron microphotographs of polymer nanocomposite **3**.

According to transmission electron microscopy data, nanocomposites **3** and **4** contain large spherical particles with sizes of 300–500 nm saturated with copper nanoparticles, which is in good agreement with the data from dynamic light scattering (Figure 8).

**Figure 9:**
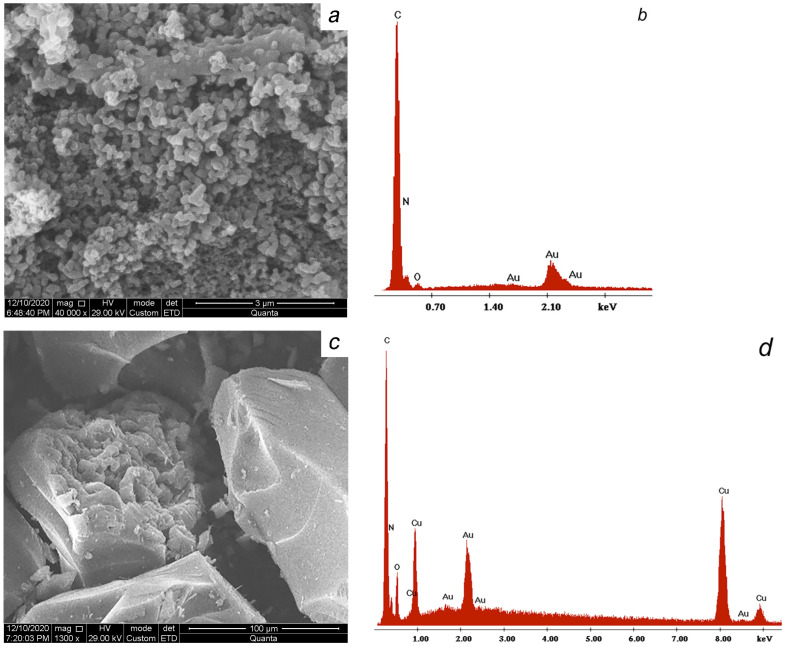
SEM (**a**,**c**) and EDS (**b**,**d**) of PVI (**a**,**b**) and nanocomposite **4** (**c**,**d**).

SEM images of the synthesized PVI and nanocomposite with CuNPs evidence their different surface morphologies (Figure 9). According to the data of scanning electron microscopy, the PVI has a highly developed fine-grained surface structure with granules 100–200 nm in size (Figure 9a). At the same time, the surface of nanocomposites has a denser structure with enlarged granules (Figure 9c). According to the EDS analysis, the elemental composition of different parts of the PVI surface is identical, which indicates the homogeneity of the polymer and nanocomposites (Figure 9b,d).

**Figure 10:**
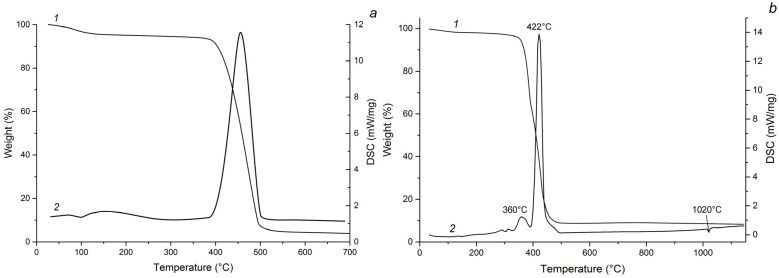
TGA (1) and DSC (2) curve for the initial poly-N-vinylimidazole (**a**) and copper nanocomposite **2** (**b**).

The resistance of PVI and nanocomposites to thermal oxidative destruction was studied by TGA and DSC methods. According to thermogravimetric analysis, the thermal stability of the initial poly-N-vinylimidazole is 380 °C (Figure 10a). Complete combustion of PVI occurs at 530 °C.

**Figure 11:**
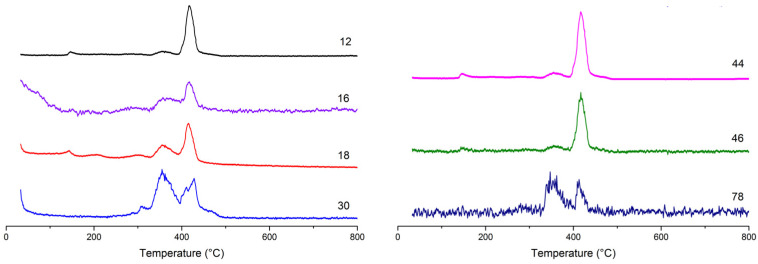
Mass spectra of copper nanocomposite **2**.

Thermal decomposition of nanocomposites **1**–**4** differs from the decomposition of the initial polymer. At 50–150 °C, the adsorbed water is released, as evidenced from the appearance of a signal with a mass number of 18 in the mass spectrum, with the weight loss being 3% (Figure 10b). At the next stage, at 350–395 °C, the weight loss of the sample is 31%, and a weak exothermic effect (maximum at 360 °C) is observed. At this stage, the polymer chains involved in the coordination of copper decompose with the release of NO and NO_2_. The mass spectra show the presence of fragments with mass numbers of 18, 30, and 46. Benzene is also formed, with a mass number of 78 (maximum at 348 °C). The last stage of polymer destruction occurs at 400–480 °C (weight loss is 40%) with an exothermic effect (maximum at 422 °C). At this stage, the carbon skeleton of the main polymer chain and imidazole groups of the polymer is burned out and the decomposition products are oxidized to form C, NO, NO_2_, C_2_H_6_, and CO_2_ (*m/z* 12, 30, 46, and 44, respectively) (Figure 11). In the differential scanning calorimetry curve, the endothermic effect responsible for the melting of metallic copper is detected at 1020 °C.

The decrease in the thermal stability of the nanocomposite, in comparison with the initial polymer, is probably a result of catalytic properties of CuNPs, which manifest themselves as a decrease in the activation energy of thermal destruction and oxidation of the polymer matrix.

The electrical conductivity of nanocomposites **1**–**4** (10^–5^–10^–7^ S/cm) is 5–7 orders of magnitude higher than the PVI polymer (1.1·10^–12^ S/cm). This is probably due to the contribution of individual local currents induced between electroconductive nanoparticles densely located in the dielectric polymer matrix. Thus, nanocomposites with CuNPs exhibit the properties of organic high-resistance semiconductors.

The presence of PVI in the reaction mixture promotes the coordinated interaction of CuNPs with imidazole rings (at the reduction stage). This ensures a homogeneous distribution of CuNPs throughout the polymer matrix and prevents their further agglomeration. The aqueous solutions of nanocomposites CuNPs **1**-**4** show no signs of sedimentation within 3 months of exposure to air at room temperature. This indicates that the CuNPs synthesized in this polymer matrix are stable and the hydrophilic PVI has high stabilizing ability.

## 4. Conclusions

New stable polymer nanocomposites with copper nanoparticles incorporated into the poly-N-vinylimidazole matrix (Mw 23.5 kDa, *PDI* 1.28) have been synthesized and characterized. The use of non-toxic PVI as a stabilizing matrix and the use of ascorbic acid as a reducing agent are consistent with the principles of green chemistry. It was found that the initial ratio of polymer and precursor of copper nanoparticles has effect on their size and polydispersity. A decrease in the copper content leads to the formation of a nanocomposite with the smallest distribution of nanoparticles in size. An increase in the copper content in nanocomposites from 1.8% to 12.3% leads to an increase in the average hydrodynamic diameters of macromolecular coils from 193 to 445 nm. New water-soluble nanocomposites with CuNPs in a PVI matrix are promising materials for the design of novel non-toxic hydrophilic antiseptics and antimicrobial components for medical purposes.

## Figures and Tables

**Table 1:** Composition and characteristics of the nanocomposites with CuNPs **1–4**.

Nanocomposite	PVI:Cu(II), mol	Yield, %	Cu Content, %wt	λ_max_, nm	Nanoparticle Size, nm	Average Hydrodynamic Diameter, nm	
						Water	Aqueous Salt Solution
**1**	40:1	85.6	1.8	556	2–8	193	17
**2**	20:1	83.1	3.5	557	2–10	269	40
**3**	10:1	85.2	6.7	535	2–12	341	110
**4**	5:1	84.5	12.3	539	6–20	445	290

**Table 2:** Average size and polydispersity of nanoparticles in nanocomposites **1**–**4**.

Nanocomposite	*D_n_*, nm	*D_w_*, nm	*PDI*
**1**	4.34	4.80	1.11
**2**	5.31	6.39	1.21
**3**	4.66	6.88	1.48
**4**	12.67	17.67	1.39

## Data Availability

Not applicable.
